# The cytochrome d oxidase complex regulated by fexA is an Achilles' heel in the *in vivo* survival of *Vibrio vulnificus*

**DOI:** 10.1080/22221751.2019.1665972

**Published:** 2019-09-23

**Authors:** Wenzhi Tan, Kwangjoon Jeong, Raghunath Pendru, Sao Puth, Seol Hee Hong, Shee Eun Lee, Joon Haeng Rhee

**Affiliations:** a Clinical Vaccine R&D Center, Chonnam National University Medical School, Hwasun-gun, Republic of Korea; b Department of Microbiology, Chonnam National University Medical School, Hwasun-gun, Republic of Korea; c College of Biology, Hunan University, Changsha, People's People’s Republic of China; d Vaxcell-Bio Therapeutics, Hwasun-gun, Republic of Korea; e Combinatorial Tumor Immunotherapy Research Center, Chonnam National University, Hwasun-gun, Republic of Korea; f Department of Pharmacology and Dental Therapeutics, School of Dentistry, Chonnam National University, Gwangju, Republic of Korea

**Keywords:** *Vibrio vulnificus*, FexA-CydAB, oxygen change, *in vivo* adaptation, *in vivo* survival

## Abstract

*Vibrio vulnificus* is a halophilic estuarine bacterium causing severe opportunistic infections. To successfully establish an infection, *V. vulnificus* must adapt to redox fluctuations in vivo. In the present study, we show that deletion of *V. vulnificus* fexA gene caused hypersensitivity to acid and reactive oxygen species. The *ΔfexA* mutant exhibited severe in vivo survival defects. For deeper understanding the role of *fexA* gene on the successful *V. vulnificus* infection, we analyzed differentially expressed genes in *ΔfexA* mutant in comparison with wild type under aerobic, anaerobic or in vivo culture conditions by genome-scale DNA microarray analyses. Twenty-two genes were downregulated in the *ΔfexA* mutant under all three culture conditions. Among them, *cydAB* appeared to dominantly contribute to the defective phenotypes of the *ΔfexA* mutant. The *fexA* deletion induced compensatory point mutations in the *cydAB* promoter region over subcultures, suggesting essentiality. Those point mutations (P_cyd_SMs) restored bacterial growth, motility, cytotoxicity ATP production and mouse lethality in the *ΔfexA* mutant. These results indicate that the *cydAB* operon, being regulated by *FexA*, plays a crucial role in *V. vulnificus* survival under redox-fluctuating in vivo conditions. The FexA-CydAB axis should serve an Achilles heel in the development of therapeutic regimens against *V. vulnificus* infection.

## Introduction

*Vibrio vulnificus* is a halophilic estuarine bacterium that causes opportunistic infection with high mortality [1]. To establish successful infections *in vivo*, *V. vulnificus* should adapt to environmental changes [2]. In the human body, different tissues are supplied with varying concentrations of O_2_, depending on their specific metabolic demands. The intestinal tissue faces daily fluctuations in perfusion and the gastrointestinal tract is characterized by a steep oxygen gradient across the epithelial layer [3]*.* Oxygen serves an important signal molecule for the global regulation of gene expression in bacterial pathogens [4]. *V. vulnificus* is a facultative aerobe that grows best in oxygen but can also multiply in the absence of oxygen. The intestinal mucosa is the first host barrier encountered by *V. vulnificus* upon oral intake. Considering the oxygen level fluctuations in tissues, it is crucial for *V. vulnificus* to develop a redox controlling system that can monitor oxygen concentration changes and regulate global gene expression to establish successful infections.

Bacterial pathogens have evolved with complex and organized systems for sensing and maintaining O_2_ homeostasis. Bacterial adaptation to O_2_ changes occurs either via direct transcriptional control by altering the DNA binding affinity of the sensory protein or two-component signaling cascades. FNR protein, the most representative direct O_2_-sensing transcription regulator, is activated at oxygen concentrations from 0 to 5 mbar (0–0.5%), the role of which has been attributed to anaerobic adaptation [5,6]. The Arc (anoxic redox control) system is a typical two-component regulator of global gene expression in response to changing oxygen concentrations. The Arc system comprises the cytoplasmic response regulator ArcA and the transmembrane sensor kinase ArcB [7]. It has been well reported that the ArcBA two-component system globally regulates the expression of genes involved in aerobic, anaerobic and microaerobic catabolic pathways in *E. coli* [5,6,8]. As a global regulator of metabolism and respiration, dysfunction of the ArcBA system is likely to attenuate the infective nature of a pathogen.

Our group has been working on *V. vulnificus* genes responsible for *in vivo* virulence expression and survival in an attempt to identify new therapeutic targets [2,9–11]. In the present investigation, we targeted the redox adaptation system and show that deletion of *V. vulnificus fexA,* a homologue of *arcA.* The *V.vulnificus f*exA is 84% identical in amino acid sequence to *E. coli arcA* [12]. The Δ*fexA* mutant caused hypersensitivity to acid and reactive oxygen species while *fnr* deletion had no significant effect. To understand underlying mechanism of *fexA* on the *V. vulnificus* pathogenicity, we analyzed differentially expressed genes in Δ*fexA* mutant compared to wild type (WT) under aerobic, anaerobic or *in vivo* culture conditions by a microarray transcriptomic analysis. Twenty-two genes were downregulated in Δ*fexA* under all three culture conditions. We constructed deletion or site-directed mutants and tested virulence traits. Through molecular genetic characterization of those genes, we discovered the *cydAB* operon encoding cytochrome d oxidase complex as an Achilles heel of *V. vulnificus* that may be targeted for the development of new antimicrobials. The FexA-CydAB axis appeared to play a pivotal role in the adaptation to oxygen changes and energy production of *V. vulnificus* for *in vivo* survival and successful infection.

## Materials and methods

### Bacterial strains, plasmids, and media

Bacterial strains and plasmids used in the present study are enlisted in Supplementary Table S1. Detailed experimental procedures are in the “Supplementary information.”

### Mutant construction

The chromosomal in-frame deletion and site-directed mutants were constructed by allelic exchange [9,10,13–16]. Primers used for PCR are listed in Supplementary Table S2. Detailed experimental procedures are in the “Supplementary Information.”

### Complementation and reversion

For complementation of the mutants, DNA fragments containing WT genes and their native promoters were generated by PCR using *pfu* polymerase (Stratagene, La Jolla, CA) with CMCP6 genomic DNA as the template using primers in Supplementary Table S2. Amplified product was purified, digested and cloned into the broad host range vector pLAFR3II [16]. The resulting plasmids were transferred into the mutant strains by tri-parental mating using a conjugative helper plasmid, pRK2013 [17]. Detailed experimental procedures are in the “Supplementary Information.”

### Bacterial growth

BD GasPak^TM^ EZ Anaerobe Gas Generating Pouch System with Indicator and BD GasPak^TM^ EZ Campy Gas Generating Pouch System were used for checking the anaerobic and microaerobic growth, respectively. *In vivo* growth was assayed in the rat peritoneal cavity dialysis tube implantation model as previously described [10]. Detailed experimental procedures are in the “Supplementary Information.”

### Ethics Statement

All animal experimental procedures were approved by the Institutional Animal Care and Use Committee in Chonnam National University under protocol CNU IACUC-H-2015-44 and animals were maintained following IACUC guidelines.

### Survival assays of bacteria exposed to oxidative challenge and acidic pH

*V. vulnificus* cells were inoculated into fresh 2.5 HI broth containing 1 mM H_2_O_2_ to the final cell concentration of 1 × 10^7 ^CFU/ml and incubated at 37°C for 1 h with shaking at 200 rpm. Viable bacterial cells were determined in time course. Acid tolerance was examined as described previously with minor modifications using 10 mM sodium citrate buffer (pH 5.0) supplemented with 2% NaCl [18].

### Determination of bacterial count in the ligated ileal loop and the blood circulation

The bacterial growth in the intestine was determined by viable bacterial counting. Detailed experimental procedures are in the “Supplementary Information.”

*Microarray analysis*. Microarray analysis was performed under aerobic, anaerobic and *in vivo* growth conditions. Detailed experimental procedures are in the “Supplementary Information.”

### Intracellular ATP level determination

Intracellular ATP level of bacteria was measured by using the ATP Determination kit (Invitrogen, Eugene, OR) in accordance with the manufacturer's protocol. Detailed experimental procedures are in the “Supplementary Information.”

### LacZ reporter construction and β-Galactosidase assay

The promoter-lacZ fusions were constructed by cloning the PCR fragments containing the promoter sequences into pTL61T plasmid [19,20]. Detailed experimental procedures are in the “Supplementary Information.”

### Identification of DNA binding proteins

Proteins that bound the *cydAB* promoter region were identified as previously described [21]. Detailed experimental procedures are in the “Supplementary Information.”

### Motility and cytotoxicity assay

For the motility test, 1 µl of 10^9^ CFU/ml logarithmic growth phase bacterial suspension was inoculated onto the 2.5% NaCl HI plates solidified with 0.3% agar (Bacto agar, Difco). The plates were incubated at 37°C and zones of migration were observed after 7 h [10,14–16]. Detailed experimental procedures are in the “Supplementary Information.”

### LD_50_ determination

The 50% lethal doses (LD_50_) of the WT and mutant *V. vulnificus* were determined with normal, iron-overloaded and sucking mice as described previously [15]. Detailed experimental procedures are in the “Supplementary Information.”

### Statistical analysis

Statistical significance was evaluated using Student's *t*-test. Statistical values were calculated using Graph Pad Prism 6 or Microsoft Excel, as appropriate. All experiments were repeated three times, and results from representative experiments are shown.

## Results

### The Δ*fexA* mutant demonstrated growth defect under aerobic, microaerobic and anaerobic conditions.

**Figure 1.**
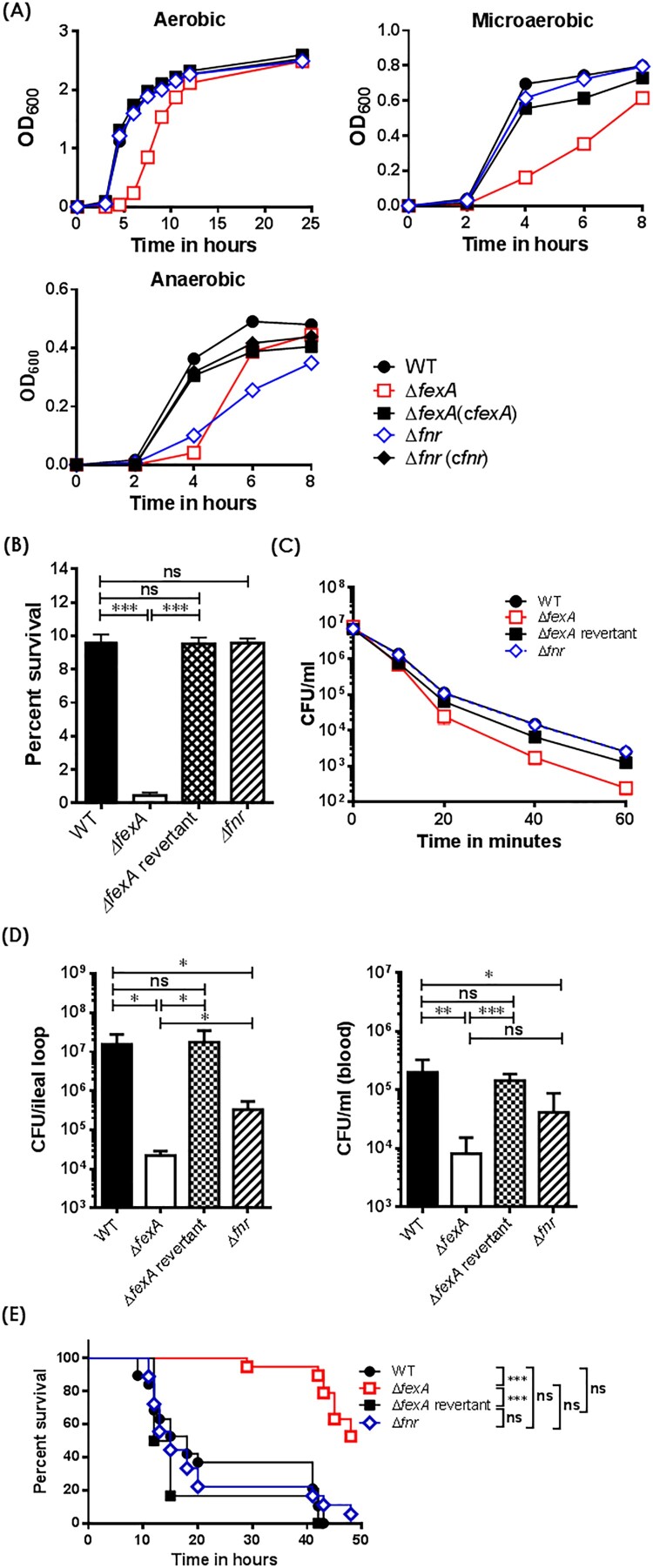
The *ΔfexA* mutant exhibit growth defect and severe deficiencies in acid tolerance, reactive oxygen resistance and *in vivo* survival. (A) Bacterial growth under aerobic, microaerobic and anaerobic conditions was determined. (B) Acid tolerance under 10 mM sodium citrate (pH 5.0) was determined as percent survival. (C) Bacterial survival in 2.5 HI broth containing 1 mM H_2_O_2_ was determined. (D) Intra-intestinal survival, growth and subsequent invasion into blood stream of *V. vulnificus* strains were determined. (E) Survival of mice intraperitoneally infected with the *V. vulnificus* strains were determined (*n* = 17 ∼18). The error bars represent standard errors. *P < 0.05; **P < 0.01, ***p < 0.001.

The Δ*fexA* manifested significantly retarded growth compared with the WT strain under aerobic, microaerobic and anaerobic culture conditions. On the other hand, the growth of the Δ*fnr* was significantly retarded only under anaerobic conditions, while no growth defects were noted under aerobic and microaerobic culture conditions. When the Δ*fexA* strain was complemented with plasmid-encoded *fexA*, the growth deficiency under aerobic conditions was fully restored (Figure 1(A)). These results indicate that FexA plays an important role in *V. vulnificus* growth under wide spectrum redox status.

### The Δ*fexA* mutant was defective in acid tolerance and reactive oxygen resistance

To compare the effect of the FexA or FNR on *V. vulnificus* virulence, we tested resistance to H_2_O_2_ and acidic pH. For this set of studies, we used a Δ*fexA* revertant instead of plasmid-based complemented strains. The complementing plasmid seemed to be unstable and easily lost under stressful conditions, such as H_2_O_2_ or acid challenge. The Δ*fexA* mutant displayed significantly decreased acid tolerance and increased susceptibility to H_2_O_2_ (Figure 1(B,C)), whereas the Δ*fnr* mutant showed WT level survival. The acid tolerance deficiency of the Δ*fexA* was fully restored in the WT revertant. The revertant strain of Δ*fexA* showed a similar survival rate to that of the WT strain (Figure 1(B,C)).

### The Δ*fexA* mutant was defective in intra-intestinal survival, growth and subsequent invasion/growth in blood stream

To test the role of FexA and FNR in the adaptation mechanism of *V. vulnificus* to oxygen fluctuation during intestinal infection, we carried out an intestinal infection experiment employing mouse ileal loop as the portal of entry. Briefly, we inoculated WT, Δ*fexA,* Δ*fexA* revertant or Δ*fnr* strain into ligated ileal loop and counted viable bacteria in the ligated ileal loop (on TCBS) and blood circulation (on 2.5 HI) to evaluate intestinal survival, growth and subsequent invasion/growth into blood stream. Viable bacteria in bloodstream at the time of counting should reflect *V. vulnficus* cells successfully invaded and grew in the bloodstream. The Δ*fexA* mutant showed defective survival in the ileal loop (*p* < 0.05) and decreased invasion/growth into the blood circulation (*p* < 0.01) compared with WT. To evaluate the role of FNR in comparison with FexA, we also tested Δ*fnr* in the same experimental setting. The Δ*fnr* mutant also showed defective survival in the ileal loop (*p* < 0.05) and decreased invasion/growth in blood circulation (*p *< 0.05). The defects of Δ*fexA* were significantly more profound than Δ*fnr* (Figure 1(D)). To further investigate the role of FexA and FNR in the gastrointestinal adaptation, we determined intragastric LD_50_ (ig LD_50_) of the WT, Δ*fexA* and Δ*fnr* mutants using suckling mice. The Δ*fexA* and Δ*fnr* mutants showed 75 and 2.3 fold increase of ig LD_50_, respectively (Supplementary Table S3). The Δ*fexA*-infected mice showed significantly delayed survival (*p* < 0.001 by Log-rank Mantel–Cox test) while the Δ*fnr* did not show significant difference (*p* > 0.05) compared with WT (Figure 1(E)). These results clearly showed that *fexA* plays more dominant roles in adapting oxygen fluctuation *in vivo* and subsequent successful intestinal infection. Consequently, we focused on the role of FexA in the *V. vulnificus* gene expression to address the defects.

### Differentially expressed genes in the Δ*fexA* strain under aerobic, anaerobic and *in vivo* conditions were identified by a DNA microarray analysis

**Figure 2.**
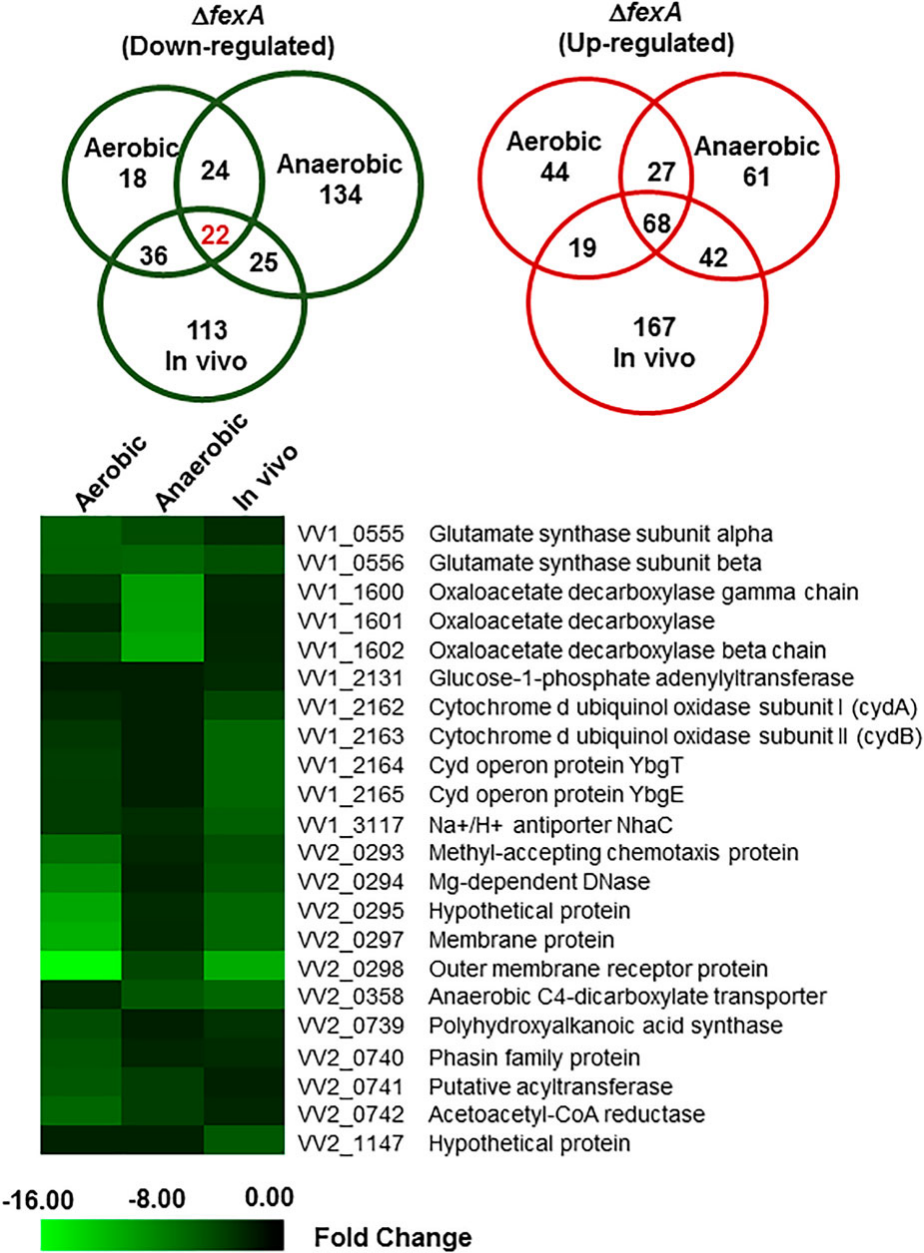
Differentially expressed genes of the Δ*fexA* strain under aerobic, anaerobic and in vivo conditions were identified by DNA microarray analysis. Venn diagram showing the extent of overlapping genes that are differentially upregulated or downregulated in the Δ*fexA* mutant among aerobic, anaerobic and *in vivo* growth conditions. Downregulated genes of the Δ*fexA* strain under aerobic, anaerobic and *in vivo* conditions.

In an effort to understand the underlying mechanism of the virulence deficiency of the Δ*fexA* strain*,* we carried out genome-scale transcriptome analysis using a DNA microarray in comparison with the WT strain under aerobic, anaerobic and *in vivo* growth conditions. By focusing on the ORFs that changed by 2-fold or more, we found that the expression of 800 (18.1% of the genome) genes was significantly affected by the *fexA* mutation under aerobic, anaerobic or *in vivo* growth conditions. The differentially expressed genes in the Δ*fexA* strain were classified into clusters of orthologous groups (COGs) for functional analyses (Supplementary Figure S1). Change in the gene expression pattern was most distinct in in vivo cultures from other culture conditions, while *in vitro* aerobic and anaerobic cultures showed less variation. Genes associated with transcription, cellular process and signaling and metabolism were most differentially regulated in the Δ*fexA* background (Supplementary Figure S1). Among the differentially regulated ORFs, 68 and 22 genes were consistently up- and downregulated, respectively, in the Δ*fexA* under all growth conditions (Figure 2). These genes would be defined to belong to core FexA regulon. In the present study, we primarily focused on 22 genes that were commonly downregulated in the absence of FexA (Table 1). These 22 genes were located in 9 loci, 5 in chromosome 1 and 4 in chromosome 2. These genes should play axial roles in the FexA regulon.

**Table 1.** Genes down-regulated in ΔfexA strain under aerobic, anaerobic and in vivo conditions (cutoff: fold change 2.0).

Locus tag	Annotated function (gene name)	COG	Fold change			Type of mutants	P*
			Aerobic	Anaerobic	In vivo		
VV1_0555	Glutamate synthase subunit alpha	E	−6.13	−5.27	−3.87	Del	NS
VV1_0556	Glutamate synthase subunit beta	E	−6.13	−6.68	−4.60	Del	NS
VV1_1600	Oxaloacetate decarboxylase gamma chain	G	−3.91	−10.30	−2.90	Del	NS
VV1_1601	Oxaloacetate decarboxylase	G	−3.63	−10.15	−2.51	Del	NS
VV1_1602	Oxaloacetate decarboxylase beta chain	G	−4.21	−10.97	−2.31	Del	NS
VV1_2131	Glucose-1-phosphate adenylyltransferase	G	−2.01	−2.17	−3.44	Del	NS
VV1_2162	Cytochrome d ubiquinol oxidase subunit I (*cydA*)	C	−3.00	−2.00	−4.16	SDM	SD**
VV1_2163	Cytochrome d ubiquinol oxidase subunit II (*cydB*)	C	−3.86	−2.22	−6.35	SDM	SD**
VV1_2164	Cyd operon protein YbgT	C	−4.02	−2.11	−6.26	–	–
VV1_2165	Cyd operon protein YbgE	C	−3.97	−2.02	−6.31	–	–
VV1_3117	Na+/H+ antiporter NhaC	P	−3.92	−3.62	−6.07	Del	NS
VV2_0293	Methyl-accepting chemotaxis protein	X	−7.36	−2.22	−4.62	Del	NS
VV2_0294	Mg-dependent DNase	X	−8.73	−2.50	−5.25	Del	NS
VV2_0295	Hypothetical protein	X	−10.46	−2.82	−7.10	Del	NS
VV2_0297	Membrane protein	X	−11.45	−2.94	−7.17	Del	NS
VV2_0298	Outer membrane receptor protein	X	−15.86	−4.51	−11.41	Del	NS
VV2_0358	Anaerobic C4-dicarboxylate transporter	T	−3.01	−5.27	−6.92	Del	NS
VV2_0739	Polyhydroxyalkanoic acid synthase	G	−4.38	−2.04	−3.68	Del	NS
VV2_0740	Phasin family protein	-	−5.16	−2.62	−3.33	Del	NS
VV2_0741	Putative acyltransferase	G/I/E	−5.88	−3.97	−2.60	Del	NS
VV2_0742	Acetoacetyl-CoA reductase	G	−6.22	−3.92	−2.63	Del	NS
VV2_1147	Hypothetical protein	X	−2.54	−2.50	−5.83	Del	NS

Notes: COG, Clusters of Orthologous Groups (COGs) category as defined at http://www.ncbi.nlm.nih.gov/COG; P*, Virulence-related phenotype of the mutant; Del, deletion mutant; SDM, site-directed mutant; NS, non-significant; SD**, significant defect; COG abbreviations; C Energy production and conversion, E Amino acid transport and metabolism, G Carbohydrate transport and metabolism, I Lipid transport and metabolism, P Inorganic ion transport and metabolism, T Signal transduction mechanisms, X Not recorded in COG.

### FexA-CydAB axis is essential for *V. vulnificus* survival

To investigate the pathogenic role of the 22 downregulated genes, we constructed in frame deletion or site-directed mutants and tested virulence changes. Notably, no significant virulence change was observed in the deletion mutants of 8 loci except for the cytochrome d ubiquinol oxidase operon (Table 1). Because the deletion of *cyd* operon proved to be lethal, we constructed site-directed *cydB* mutants by changing amino acids in the highly conserved positions and determined bacterial growth under aerobic condition. As shown in Supplementary Figure S2, the *cydB* R100H and *cydB* G144A mutants showed severe growth retardation even compared with the Δ*fexA* mutant. These results demonstrate that the cytochrome d oxidase complex under FexA regulation is essential for survival.

### The Δ*fexA* mutant induced spontaneous compensatory point mutations in the *cydAB* promoter region

The Δ*fexA* formed smaller colonies on 2.5 HI plates. Interestingly, when the mutant was revived from frozen stocks, large colony variants appeared on agar (Supplementary Figure S3, arrow). When *cydAB* gene sequences were determined in the large colony Δ*fexA* revertants, single nucleotide mutations in the *cydAB* promoter region were noted. We found four types of point mutations: A to G at −105 bp (P_cyd_SM4), C to A or T at −109 bp (P_cyd_SM2, P_cyd_SM3), and A to C at −117 bp (P_cyd_SM1), as shown in Supplementary Figure S3. The mutation sites are located inside or 5 bp upstream of the second putative FexA binding site.

### Compensatory mutations in *cydAB* promoter region (PcydSMs) significantly enhanced *cydAB* expression

**Figure 3.**
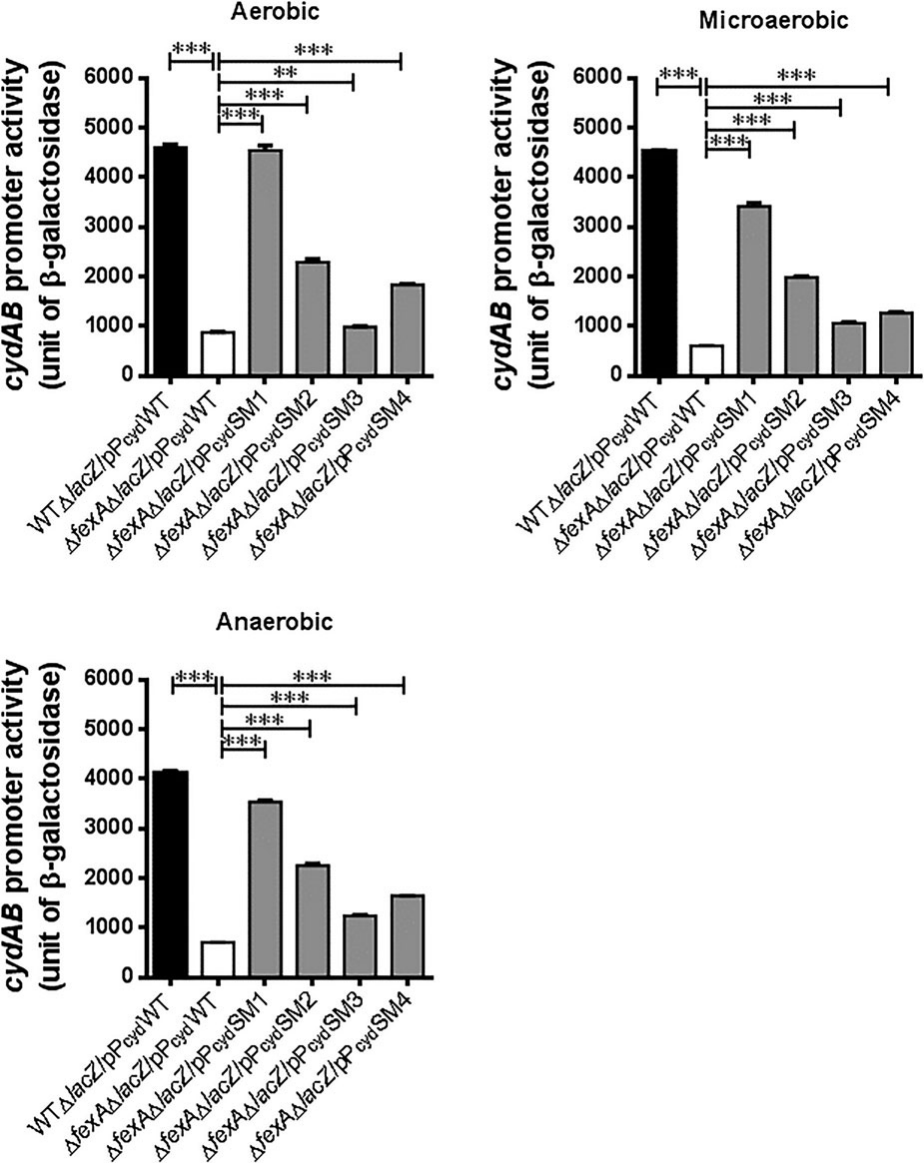
Compensatory mutations in the *cydAB* promoter region significantly enhanced cydAB promoter activity. The *cydAB* promoter activities were determined by measuring β-galactosidase activity of the *cydAB-lacZ* reporter plasmids under aerobic, microaerobic and anaerobic growth conditions. The error bars represent standard errors. ***p < 0.001.

To confirm whether the single nucleotide mutations in the *cydAB* promoter region (P_cyd_SM1, P_cyd_SM2, P_cyd_SM3 and P_cyd_SM4) affected *cydAB* transcription in the Δ*fexA* mutant background, we constructed five types of *cydAB*-*lacZ* reporter plasmids by using the pTL61T vector (pP_cyd_WT, pP_cyd_SM1, pP_cyd_SM2, pP_cyd_SM3 and pP_cyd_SM4) and the resulting reporter plasmids were transformed into Δ*fexA*Δ*lacZ* and WTΔ*lacZ* strains (Supplementary Table S1). For this experiment, microaerobic culture was used to mimic the intestinal environment where *V. vulnificus* starts infection [22]. As shown in Figure 3, in the *ΔfexA* mutant background, the promoter activities of P_cyd_SM1, P_cyd_SM2, P_cyd_SM3 and P_cyd_SM4 were significantly enhanced compared to those WT *cydAB* promoter (pP_cyd_WT) under *in vitro* aerobic, microaerobic and anaerobic conditions (*p *< 0.001; Figure 3). Among the single nucleotide mutant promoter reporter, P_cyd_SM1 showed the highest expression under tested conditions in the Δ*fexA* background. The activation level of these mutated *cydAB* promoters in the WT background were similarly enhanced in all four types of mutations, especially under aerobic and microaerobic conditions (Supplementary Figure S4).

### CydAB play a critical role in fexA-mediated regulation of bacterial growth and virulence expression

**Figure 4.**
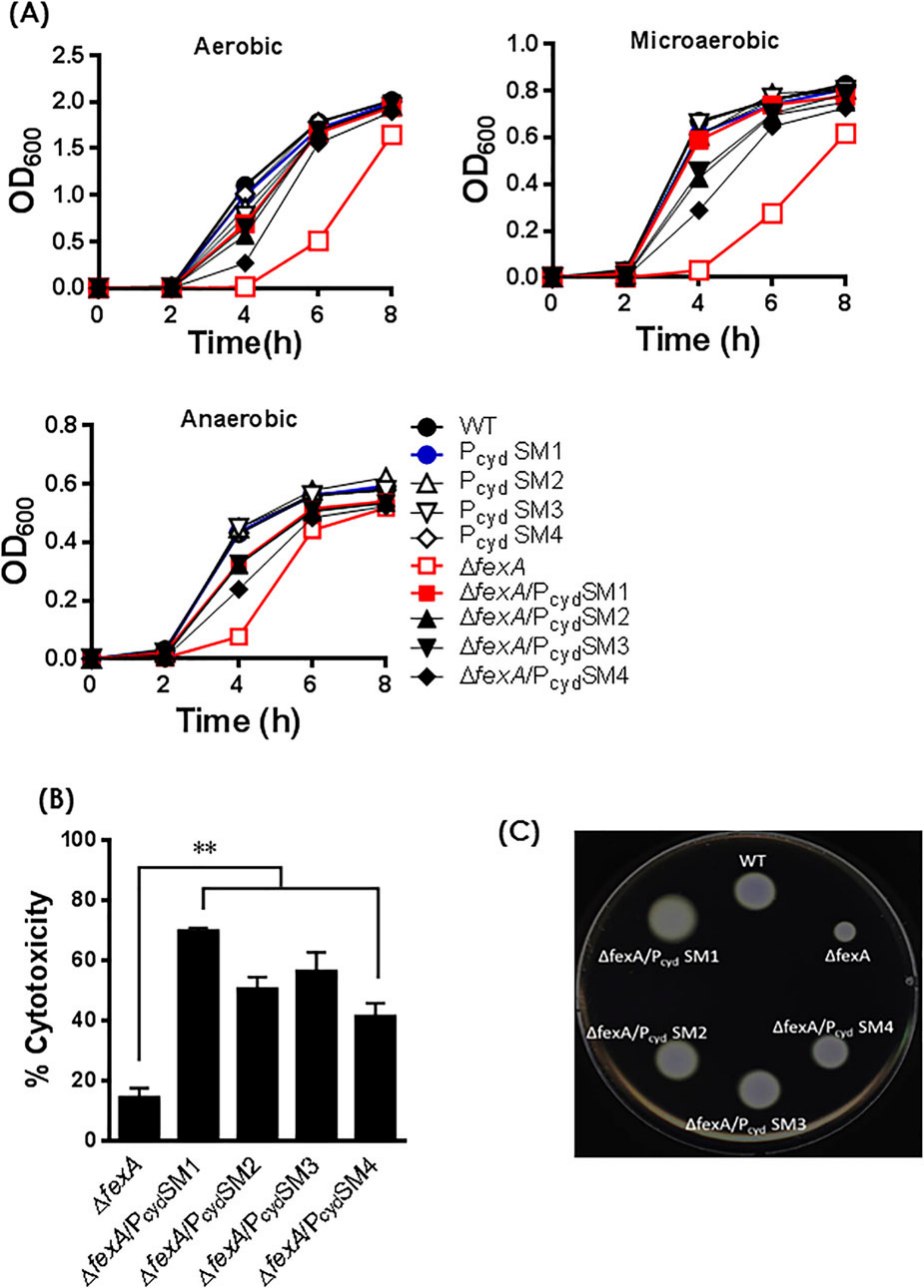
Compensatory mutations in the *cydAB* promoter region reversed phenotypic changes of the *ΔfexA* mutant. (A) Bacterial growth was monitored by measuring the OD_600_ value of cultures at different time points. Cytotoxicity (B) and motility (C) of the WT strain and respective mutants were determined. The error bars represent standard errors **p < 0.01.

To address whether the growth retardation and virulence defect of the Δ*fexA* mutant was due to decreased expression of *cydAB,* we constructed Δ*fexA/*P_cyd_SMs strains by swapping the WT *cydAB* promoter with compensatory mutated ones (P_cyd_SMs) and tested bacterial growth, cytotoxicity and motility of the strains. The bacterial growth retardation (Figure 4(A)), cytotoxicity deficiency (Figure 4(B)) and motility defect (Figure 4(C)) of the Δ*fexA* mutant was restored in Δ*fexA/*P_cyd_SMs strains. These results corroborate that compensatory mutations in the *cydAB* promoter region restored the growth retardation, cytotoxicity and motility of the *ΔfexA* mutant.

### The FexA-CydAB axis modulates energy production of *V. vulnificus*

**Figure 5.**
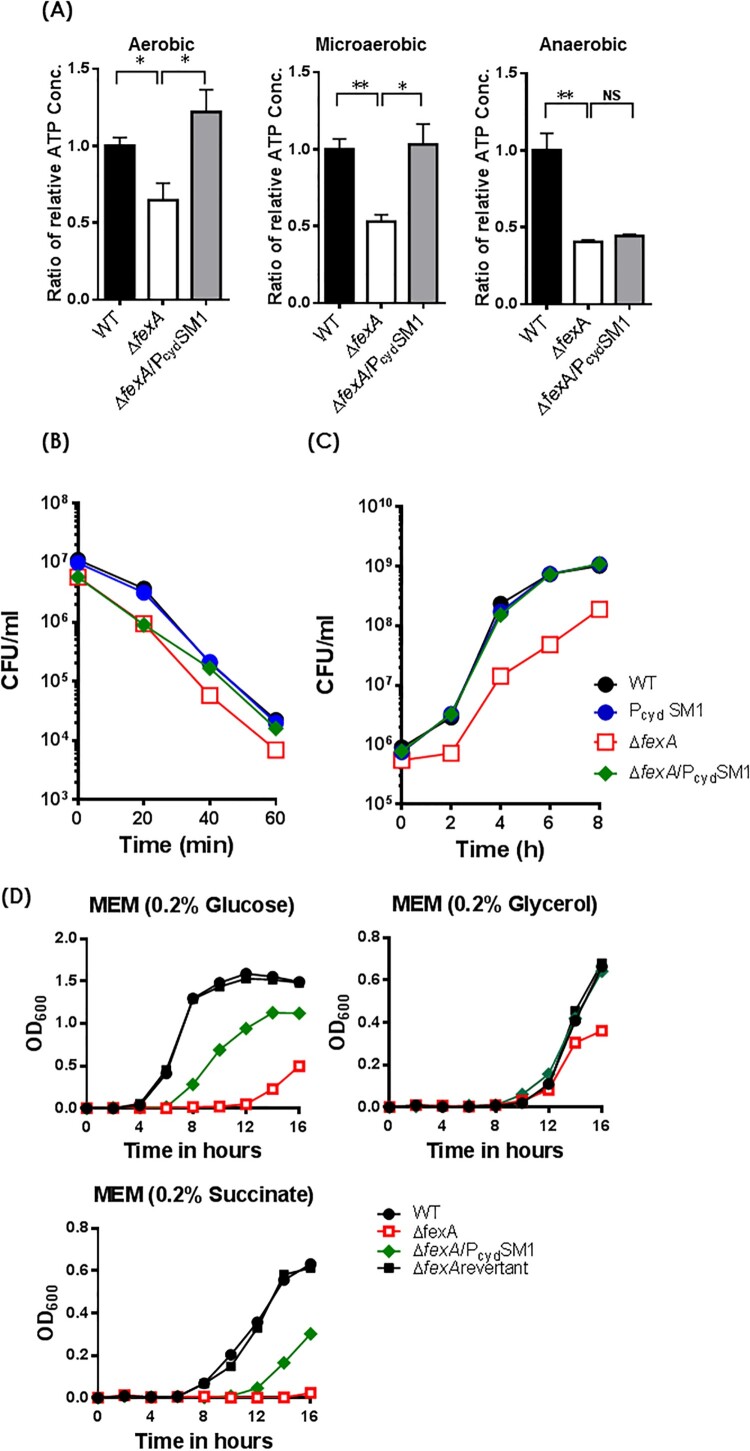
Compensatory mutations in the *cydAB* promoter region restore ATP production and the deficiencies of H_2_O_2_ resistance and *in vivo* survival of *ΔfexA* mutant. (A) Intracellular ATP production was measured under aerobic, microaerobic and anaerobic growth conditions. (B) Bacterial survival in 2.5 HI broth containing 1 mM H_2_O_2_ was determined. (C) *In vivo* growth of the *ΔfexA* mutant in the rat peritoneal cavity (n = 4) was determined by using a dialysis tube implantation model. (D) Bacterial growth in minimal essential medium (MEM) supplemented with 0.2% glucose, 0.2% glycerol or 0.2% succinate under aerobic culture condition was determined. The error bars represent standard errors. *p < 0.05; **p < 0.01; ns, not significant.

Given that cytochrome d oxidase is the sole terminal oxidase in the electron transport chain of *V. vulnificus* and *cydAB* expression is downregulated in the *ΔfexA* mutant*,* we hypothesized that *ΔfexA* can produce less amount of ATP and P_cyd_SM1 replacement would restore the ATP production. As expected, the intracellular ATP level of the Δ*fexA* mutant grown under aerobic, microaerobic and anaerobic conditions was significantly lower than that of the WT strain, which was restored by introduction of the four types of mutations in the promoter region (Figure 5(A)). These results explain that the low levels of intracellular ATP in the Δ*fexA* mutant resulting from decreased expression of *cydAB* contribute to the growth and virulence deficiencies under, at least, aerobic and microaerobic conditions.

### The FexA-CydAB axis is essential for the adaptation of oxygen fluctuations and *in vivo* survival of *V. vulnificus*

Introduction of the P_cyd_SM1 mutation in the *cydAB* promoter region of the Δ*fexA* strain also fully restored its resistance to H_2_O_2_ (Figure 5(B)) and *in vivo* growth (Figure 5(C)), as well as the LD_50_s of the Δ*fexA*/P_cyd_SM1 strain to the level of the WT strain in normal i.p., iron-overloaded i.p., and suckling mouse i.g. infection (Table 2). These results indicate that the FexA-CydAB axis dominantly modulates energy production in infecting *V. vulnficus* in response to variable *in vivo* oxygen tension for their survival and virulence expression. To further understand the regulation of FexA on the *cydAB* in the context of metabolism, we further determined bacterial growth in a minimal essential medium (MEM) supplemented with different carbon sources such as glucose, glycerol, or succinate (a non-fermentable carbon source). In contrast to the wild type, the Δ*fexA* mutant manifested delayed growth and was completely defective growing in 0.2% succinate MEM media (non-fermentable carbon source). In addition, the growth defects of Δ*fexA* mutant were fully recovered in the *fexA* revertant in all culture conditions and a compensatory point mutation in the *cydAB* promoter region (Δ*fexA*/P_cyd_SM1) restored bacterial growth in 0.2% succinate MEM media (Figure 5(D)). These results corroborate that FexA directly regulates *cydAB*, critical components of complex III in the electron transfer system of *V. vulnificus* using succinate for proton generation and that the FexA plays a pivotal role in *in vivo* proliferation of *V. vulnificus* via regulation of expression of the *cydAB*.

**Table 2.** Effect of mutations on the lethality of *V. vulnificus* in mice.

Strain	LD_50_ (Fold increase in LD_50_: Mutant/Wild type)		
	Intraperitoneal normal mice	Intraperitoneal iron-overloaded mice	Intragastric sucking mice (6 days)
Wild type	4.0 × 10^5^	1.0	4.0 × 10^6^
Δ*fexA*	1.0 × 10^7^ (25)	6.6 × 10^6^ (660,000)	3.0 × 10^8^ (75)
Δ*fexA/*PcydSM1	4.0 × 10^5^ (1)	1.0 (1)	1.0 × 10^7^ (2.5)

### The P_cyd_SM1 compensatory mutation enhanced the *cydAB* promoter and *fexA* binding

**Figure 6.**
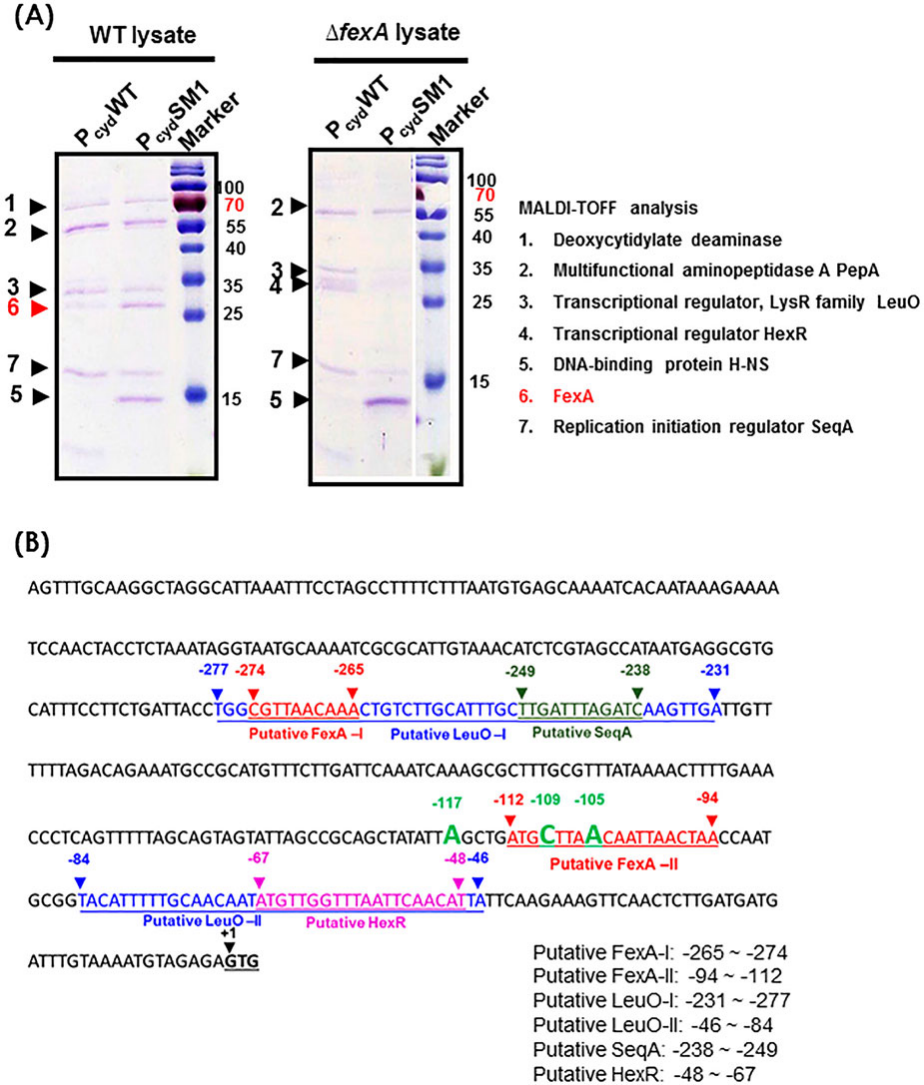
Analysis of the *cydAB* promoter-binding protein complex. (A) Determination of the *cydAB* promoter binding proteins. A biotin-labeled DNA fragment of the *cydAB* promoter region was affixed to streptavidin-conjugated Dynabeads, and then incubated with *V. vulnificus* cytoplasmic extract. Non-adhering and low-specificity DNA-binding proteins were removed by repeated washing and DNA-binding proteins were eluted. Single protein bands were cut from the SDS-PAGE gel for MALDI-TOF Mass Spectrometry assay. (B) The promoter region of the *cydAB* operon. Bioinformatic analysis suggests two putative FNR binding (blue colour), two putative FexA binding (red colour), two LeuO binding (highlighted), one HexR (green colour) and one SeqA (violet colour) sites.

To further address the role of FexA on the *cydAB* promoter and determine why compensatory point mutations in the *cydAB* promoter region enhanced *cydAB* transcription, we analyzed the components of the P_cyd_-protein complex by employing a biotin bead-based precipitation assay as described in Material and Method section. As shown in Figure 6, the WT *cydAB* promoter (P_cyd_WT) was bound by five proteins under aerobic culture conditions; FexA, deoxycytidylate deaminase, PepA, LeuO, and SeqA. Notably, P_cyd_SM1 compensatory point mutation significantly increased the binding of FexA protein to the *cydAB* promoter region. When the Δ*fexA* mutant lysate was incubated with the P_cyd_WT DNA fragment, PepA, LeuO, SeqA and transcriptional regulator HexR were identified (Figure 6(A,B)). Notably, P_cyd_SM1 bound to H-NS, while P_cyd_WT did not. In the P_cyd_ promoter region, multiple T-N_11_-A sequences were observed. Among them, two sites (−231 ∼ −277 bp and −46 ∼ −84 bp) were predicted to be putative LeuO binding sites and overlapped the putative FexA (−265 ∼ −274 bp), FNR (−236 ∼ −249 bp), SeqA (−238 ∼ −241 bp) and HexR (−48 ∼ −67 bp) binding sites. These results suggest that the FexA protein binds to the P_cyd_ promoter and counteracts probable transcriptional repressors to promote expression. In the absence of FexA, compensatory single nucleotide mutations, such as P_cyd_SM1 confering a lowered binding affinity to transcription repressors, should lead to the expression of crucial survival genes.

## Discussion

For the successful infection, opportunistic pathogens must adapt to redox fluctuations *in vivo.* The ArcA and FNR systems have been reported to be involved in the fine-tuning of metabolism in response to oxygen fluctuations in various bacteria [8,23–30]. In *V. vulnificus*, deletion of *fexA*, an *arcA* ortholog, attenuated a plethora of *V. vulnificus* functions, including *in vitro* growth and *in vivo* survival, resistance to reactive oxygen species (ROS) and acidic pH, motility, cytotoxicity and mouse lethality, all of which contribute to pathogenicity *in vivo*. A whole transcriptome analysis of the WT and *ΔfexA* under aerobic, anaerobic and *in vivo* conditions provided a glimpse of the *V. vulnificus* FexA regulon. In the FexA regulon, terminal cytochrome d oxidases (*cydAB*) play critical roles for survival and virulence expression of *V. vulnificus* in response to oxygen availability changes in the host environment. The *cydAB* expression decreased in the Δ*fexA* strain under all of the tested culture conditions and deletion of *cydAB* gene resulted in lethal mutation. The Δ*fexA* formed smaller colonies on 2.5 HI plates compared to WT. Notably, some Δ*fexA* colonies reverted to its original size by spontaneous transversion point mutations in the *cydAB* promoter region. The compensatory point mutations in the *cydAB* promoter region significantly enhanced its expression level in WT and the Δ*fexA* mutant. Furthermore, a compensatory point mutation, P_cyd_SM1, reversed all attenuated phenotypes of the Δ*fexA.* The FexA protein was detected in the *cydAB* promoter-bound protein complex and the P_cyd_SM1 compensatory mutation enhanced the cydAB promoter-FexA binding. Conclusively, we presume that the terminal cytochrome d oxidases are Achilles heel of *V. vulnficus* and may serve as potential therapeutic targets against *V. vulnificus*.

*E. coli* has two cytochrome oxidase complexes, cytochrome d oxidase and cytochrome o oxidase (*cyoABCDE*), to generate the proton motive force for ATP biosynthesis. However, *V. vulnificus* CMCP6 does not harbour any homologues of *E. coli cyoABCDE.* Accumulating reports have shown that inactivation of redox regulators or the terminal oxidase induce negative effect on bacterial stress resistance and survival under *in vivo* conditions [31–34]. Given that CydAB is the sole cytochrome oxidase complex and deletion of *cydAB* was lethal to *V. vulnificus* (Table 1), the *cydAB* is indispensable for the energy production under the control of FexA during *V. vulnificus* infection.

**Figure 7.**
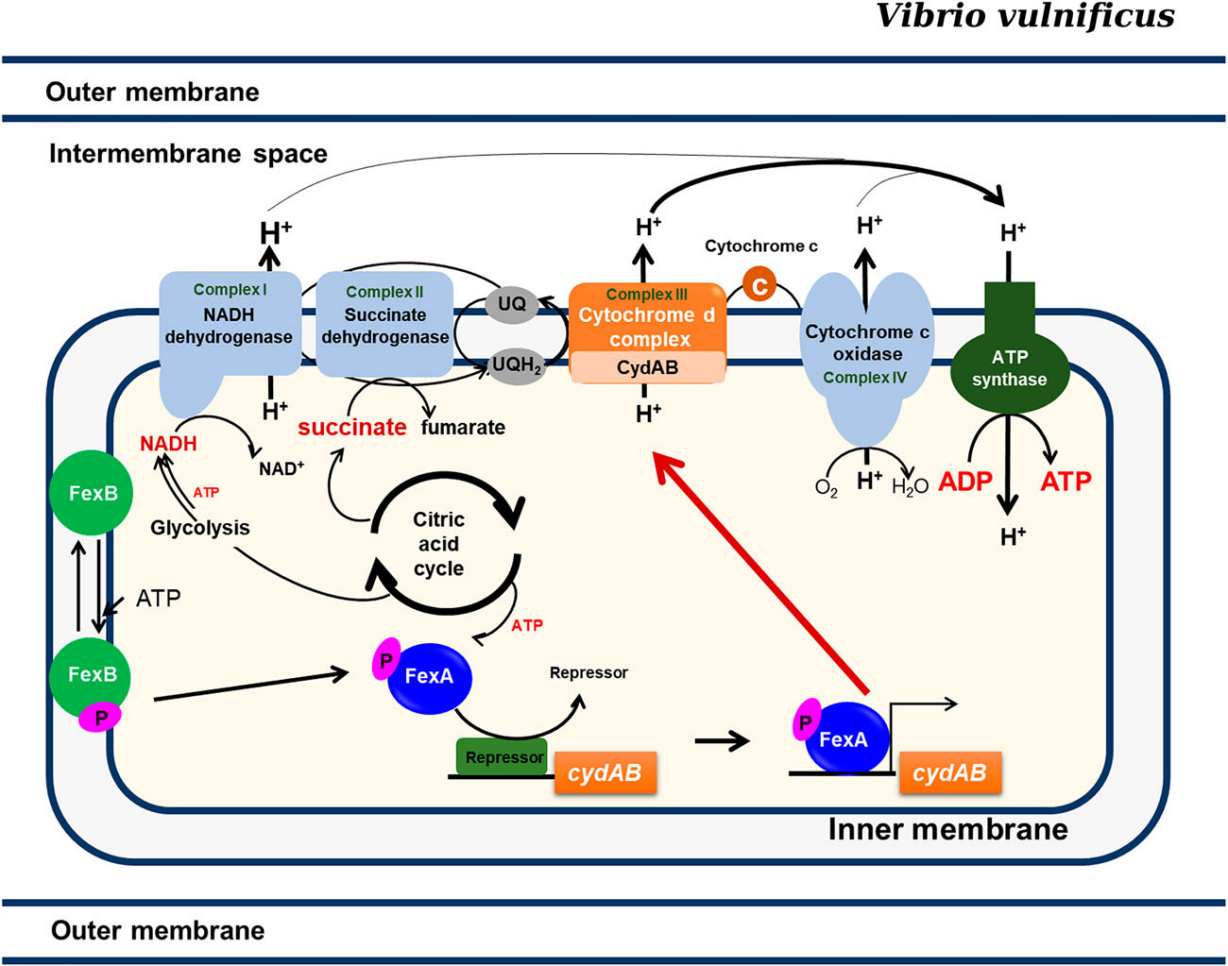
The FexAB two component system regulates the adaptive responses to oxygen availability. Upon stimulation, FexB undergoes autophosphorylation, and a phosphoryl group is transferred to FexA by a His-Asp-His-Asp phosphorelay, which consequently activates the expression of *cydAB. CydAB* encodes the terminal oxidase of the electron transport pathway, which is essential for the survival of *V. vulnificus.* The most important role played by FexA in *V. vulnificus* is the activation of *cydAB* expression.

In the present study, based upon transcriptome data and KEGG database, we present how the energy would be generated by the FexA-CydAB axis *in vivo* (Figure 7). It is predicted that at least three enzymatic complexes in the cytoplasmic membrane would generate proton gradient contributing to ATP production. The KEGG database predicts that NADH dehydrogenase, CydAB, and cytochrome c oxidase will generate protons (www.genome.jp/kegg-bin/show_pathway?vvu00190+VV1_2074). Notably, only the CydAB operon in the electron transfer chain was under the control of FexA and a deletion mutation of the NADH dehydrogenase (VV1_2074) in the first complex could be constructed (data not shown), suggesting a differential role in the electron transfer chain. Redox state maintenance is a key cellular function and is crucial for bacterial adaptability. Compromised redox homeostasis should be detrimental to the survival of bacteria. Many reports addressed that the redox imbalance caused by inactivation of redox regulators or the terminal oxidases has a profound negative effect on bacterial stress resistance and survival *in vivo* [31–34]. Considering the non-existence of the cytochrome o oxidase operon in the *V. vulnificus* genome, the cytochrome d oxidase CydAB should never be dispensable for the survival of *V. vulnificus*, not like other bacteria that harbour both of them, in which *cydAB* orthologs could be deleted. In addition to *cydAB*, we also found that multiple genes potentially associated with redox regulation under FexA regulation (Table 1). We have constructed deletion mutants for those genes and analyzed their roles in *V. vulnificus* pathogenicity. Among them, CydAB was the most dominant.

Bacterial motility is highly energy-consuming and the polar vibrio flagellar motor is driven by a Na^+^ motive force at the expense of ATP [35]. Considering that the expression of flagellar biosynthesis genes was not affected by the FexA mutation and that the motility deficiency of the Δ*fexA* mutant was fully recovered by increasing *cydAB* expression, the decreased motility should have contributed to the adhesion defects of Δ*fexA*. The most potent cytotoxin RtxA1 is expressed upon the close encounter of *V. vulnificus* with host cells, which is driven by flagellar motility and subsequent adhesion to host cell surface [9,36]. Decreased adhesion should have resulted in the cytotoxicity defect.

## Supplementary Material

Supplemental Material
