# Supplementary material for: The cytochrome d oxidase complex regulated by fexA is an Achilles' heel in the in vivo survival of Vibrio vulnificus
Source: Emerg Microbes Infect. 2019 Sep 23;8(1):1406–15. doi: 10.1080/22221751.2019.1665972 (PMC6764401; doi:10.1080/22221751.2019.1665972)
Supplement: Supplemental Material [file TEMI_A_1665972_SM7598.zip › R1_FexA_suppl_information_FINAL_091519_clean.docx]

**Supplementary Figure Legends**

**Supplementary Figure 1. Differentially expressed genes of the ∆*fexA* strain under aerobic, anaerobic and in vivo conditions were identified by DNA microarray analysis.** Differentially expressed genes in the ∆*fexA* mutant were classified according to Clusters of Orthologous Groups (COGs) as defined at <http://www.ncbi.nlm.nih.gov/COG> under aerobic, anaerobic and in vivo growth conditions.

**Supplementary Figure 2. Site-directed mutations of *cydB* severely affect *V.vulnificus* aerobic growth.** Bacterial growth was monitored by measuring the OD_600_ value of cultures at different time points**.** At each time point, the OD_600_ value represents the mean of two independent cultures of each strain tested.

**Supplementary Figure 3. Compensatory point mutations in the large colony variant of *ΔfexA***. Colony morphology of the WT and *ΔfexA* strains. Bacteria were grown on 2.5 HI agar plates at 37˚C. The *ΔfexA* mutant exhibited small colonies on 2.5 HI plates, large colony variants could be observed after storage at -80 ˚C. Spontaneous mutations were selected for in these large-sized colonies of the *ΔfexA* mutant, when the mutant coped with stress during the temperature shift. Schema showing the identified spontaneous single-nucleotide mutations in the *cydAB* promoter region of large colony variants of the *ΔfexA* mutant.

**Supplementary Figure 4. Compensatory point mutations in the *cydAB* promoter region significantly enhance *cydAB* expression in the wild type.** Transcription β-galactosidase activity assay of the *cydAB* promoter under aerobic, microaerobic and anaerobic growth conditions. β-galactosidase activity was produced by fusions of *lacZ* with an intact *cydAB* promoter region and its mutated derivatives in ∆*lacZ* and ∆*fexA* backgrounds. . * *p* < 0.05; ** *p* < 0.01；*** *p* < 0.001.

**Supplementary Materials and Methods**

**Bacterial strains, plasmids, and media.** *V.vulnificus* CMCP6 is a clinical isolate from a male septicemic patient, isolated at the Chonnam National University Hospital, South Korea. *V.vulnificus* CMCP6 was grown in 2.5% NaCl heart infusion (HI) medium while *Escherichia coli* strains were grown in Luria-Bertani (LB) medium supplemented appropriately with antibiotics. Bacteria were grown at 37◦C under shaking conditions (200rpm). Thiosulfate citrate bile salt sucrose agar (TCBS) (Merck, Darmstadt, Germany) was used as the selective medium for *V.vulnificus*. For *E. coli*, antibiotics were used at the following concentrations: ampicillin (Amp) 100μg/ml, kanamycin (Km) 100μg/ml, chloramphenicol (Cm) 30μg/ml, and tetracycline (Tc) 12.5μg/ml. For *V.vulnificus*, Amp 20μg/ml, Tc 2μg/ml and Cm 2μg/ml were used.

**Construction of in-frame deletion mutants and site-directed mutants.** The chromosomal in-frame deletion mutants and site-directed mutants were constructed in *V. vulnificus* using the allelic exchange method. Primers used for PCR reactions are listed in Table 2. For in-frame deletion mutant construction, upstream and downstream *ca* 1000 base pair (bp) fragments of target genes were amplified separately and cloned into pCR2.1 vector using the Original TA Cloning Kit (Invitrogen, Carlsbad, CA, USA). Fusion 2 kbp fragments of upstream and downstream were created by ligation, and were subcloned into the pDM4 suicide vector after digested with appropriate restriction enzymes. The resulting recombinant vector was transformed into *E. coli* SM10 λ pir and subsequently transferred to *V. vulnificus* CMCP6 by conjugation. Stable Cm resistant transconjugants were selected on TCBS agar plates containing Cm. Plating of the transconjugants on 2.5% NaCl HI agar plate containing 10% sucrose was done to select clones that experienced the second homologous recombination events forcing excision of the vector sequence and leaving only mutated or wild type allele of target genes. Each in-frame deletion mutation was confirmed by PCR of the chromosomal DNA from the respective mutant. The resulting mutant strains are listed in Table 1. Site-directed mutagenesis was performed with a similar approach as deletion mutant construction. The PCR fragment containing the *cydB* ORF region or promoter region of *cydAB* operon with single nucleotide substitution was generated by cross over PCR by sets of primers listed in Table 2, and was subcloned into pDM4 suicide vector for the next conjugation and screening step. The expected mutation was verified by DNA sequencing.

**Complementation and reversion.** For complementation of the mutants, DNA fragments containing wild type genes and their native promoters were generated by PCR amplification using Pfu polymerase (Stratagene, La Jolla, CA) with CMCP6 genomic DNA as the template using primers listed in Table 2. Amplified product was purified, digested and cloned into the broad host range vector pLAFR3II. The resulting plasmids were transferred into the mutant strains by triparental mating using a conjugative helper plasmid, pRK2013. The transconjugants were screened on TCBS agar plates containing appropriate antibiotics and confirmed by PCR. For the revertant strain construction, DNA fragment containing target WT gene and their upstream and downstream 1000 base pair (bp) was amplified and cloned into pDM4 suicide vector for the next conjugation and screening step. The revertant strain construction was confirmed by PCR and DNA sequencing.

**Bacterial growth *in vitro*.** To assess the growth of bacteria, overnight grown bacterial cultures in HI medium were washed twice with phosphate buffered saline (PBS, pH7.2) and inoculated into various medium, The optical density at 600nm (OD600) was measured spectrophotometerically (Ultrospec6300Pro, Amersham Biosciences) at selected time points. BD GasPakTM EZ Anaerobe Gas Generating Pouch System with Indicator and BD GasPakTM EZ Campy Gas Generating Pouch System (Becton, Dickinson and company) were used for evaluating the anaerobic and microaerobic growth, respectively. BD GasPakTM EZ Campy Gas Generating Pouch System produces an atmosphere with approximately 5-15% oxygen. Growth in nutrient-limited medium was performed in M9 minimal medium supplemented with 0.2% carbon source such as glucose, succinate or glycerol.

**Cytotoxicity assay.** HeLa cells were seeded in 24-well culture plates (Corning®, Corning, NY, USA) at a concentration of 1×10^5^ cells/ml and cultured at 37℃ and 5% CO2. After 24 hours, the cells were washed twice with 1 ml of pre-warmed serum-free DMEM. *V. vulnificus* cells at the exponential growth phase in 2.5% NaCl HI broth were harvested by centrifugation and washed twice with PBS, and resuspended in PBS to 1×10^9^ CFU/ml. The HeLa cells were infected with the bacteria at the MOI of 100. The CytoTox nonradioactive cytotoxicity assay kit (Pro-mega, Madison, WI, USA) was used to quantitate cytosolic lactate dehydrogenase (LDH) release as an indicator of cytotoxicity. The supernatants were collected at the selected time points for estimation of cytotoxicity. Thirty microliter (30 μl) of each supernatant was transferred to a 96-well plate and mixed with same volume of reconstituted LDH substrate mix. After 30 min incubation at room temperature in dark condition, 30 μl of stop solution was added to each well and the absorbance at 490 nm was measured. Complete cell lysate and PBS served as the positive and negative control, respectively.

**LD_50_ determination.** The 50% lethal doses (LD_50_) of the WT and mutant *V. vulnificus* were determined with normal and iron-overloaded mice. One milliliter of the overnight culture was inoculated into 100 ml of fresh 2.5% NaCl HI broth and then the cultures were grown at 37 °C and 200 rpm for 4.5 h. The cells were harvested by centrifugation and washed three times with PBS. The cell pellet was resuspended in PBS. Seven-week-old specific pathogen-free (SPF) female CD-1 mice (Daehan animal Co., Daejeon, Korea) were used for the experiment. Groups of five mice were inoculated intraperitoneally with 10-fold serial dilutions of test strains. Deaths were observed for 48 hrs. The intragastric 50% lethal dose (i.g. LD50) was tested using randomly bred SPF CD-1 suckling mice (Daehan Animal Co., Daejeon, South Korea). Six-day-old infant mice were administered 10-fold serial dilutions of fresh bacterial suspensions containing 0.1% Evans blue (Sigma-Aldrich Co., St. Louis, MO) to ensure correct intragastric administration. The challenged mice were monitored for 48 h.

**Survival assays of bacteria exposed to oxidative challenge and acidic pH.** *V. vulnificus* cells were grown in 2.5% NaCl-HI broth at 37℃ overnight with shaking at 200 rpm. The culture was then washed twice with PBS and inoculated into 15 ml of fresh 2.5% NaCl-HI broth containing 1 mM H_2_O_2_ to the final cell concentration of 1x10^7^ CFU/ml and incubated at 37 °C for 1 h with shaking at 200 rpm. Culture aliquots were taken at different time intervals, and viable counts were determined by plating on 2.5% NaCl-HI agar plates. For acid tolerance determination, bacterial strains were grown to OD600 0.6 to 0.8 (mid-log phase) in 2.5% NaCl HI broth, harvested, washed with 10 mM sodium citrate buffer (pH 5.0) supplemented with 2% NaCl (SCBN) and suspended in the same buffer to a final concentration of 105 CFU/ ml. Cell suspensions were incubated at 37°C with shaking (200 rpm) for 90 min. Viable cell count carried out by plating on 2.5% NaCl HI agar plates. The percentage of survivors was calculated by using the CFU/ml as determined immediately after inoculation as 100%.

**Determination of bacterial count in the ligated ileal loop and the blood circulation.** The bacterial growth in the intestine was determined as previously described [1, 2]. Briefly, seven-week-old SPF female CD-1 mice were starved for 16 h and anesthetized with a mixture of 10% zolazepam-tiletamine (Zoletil; Virbac Laboratories, France) and 5% xylazine (Rumpun; Byer Korea, South Korea) dissolved in PBS. The mouse was operated and the ileum part of the small intestine was tied off in a 5-cm segment as an ileal-loop ligation, and then V. vulnificus cells (4.0 × 10^6^ CFU/400 μl in PBS) were inoculated into the ligated segment. After 6 hours of infection, the ileal loops were cut out and chopped to release bacterial cells into PBS (1ml of PBS per loop). Further, to ensure that bacterial cells were completely released from the chopped loops, the mixture was forcefully vortexed and V. vulnificus cells in ligated ileal loops were serially diluted and counted by plating on Vibrio-selective TCBS agar plates. At the same time, the bacterial cells that translocated from the intestine to the bloodstream were also measured. The blood samples were acquired by cardiac puncture and viable bacterial cells were counted by plating on 2.5% NaCl HI agar plates.

**RNA extractions.** For the microarray analysis, total RNA from log phase *V. vulnificus* was extracted using RNeasy mini kit (QIAGEN, Germany) in accordance with the manufacturers’ protocol. Microarray analysis was performed under aerobic, anaerobic and in vivo growth conditions, wild type strain and FexA mutant were grown aerobically and anaerobically to mid-log phase in HI broth and we used dialysis tube implantation model as in vivo condition. RNA purity and integrity were evaluated by denaturing gel electrophoresis, OD260/280 ratio, and analyzed on Agilent 2100 Bioanalyzer (Agilent Technologies, Palo Alto, USA).

**Microarray, hybridization, and analysis.** Microarray analysis was performed under aerobic, anaerobic and *in vivo* growth conditions. The oligonucleotide DNA microarray chip used in this study was fabricated by Roche NimbleGen, Inc. (Madison, USA). The hybridization and its related procedures were performed by Macrogen Co. Ltd. (Seoul, Korea). To achieve a satisfactory amount of total RNA for hybridization, 10 Sprague Dawley rats (7-week-old female, DaiHan Biolink, Daejeon, Korea) were used for *in vivo* growth of *V. vulnificus* WT and the *ΔfexA* mutant. The oligonucleotide sequence of each open reading frame was designed based on the genome sequences of *V. vulnificus* CMCP6. Array data export processing and analysis were performed using NimbleScan v2.5 (Gene Expression RMA algorithm). Raw data were extracted, and a single raw intensity value was determined for each gene in each array by averaging spot 2 replicates of all 8 probes for each of the 4411 genes. Comparative analysis between WT and the mutant was carried out using the fold-change and local pooled error (LPE) test-adjusted false discovery rate (FDR) *P* value. Out of 4,411 genes spotted on the array, 3,858 genes passed the LPE test (one-way ANOVA, Tukey’s HSD test). We restricted the analyses to only include highly affected genes (i.e., those with a ratio > 2.0-fold). Statistical significance of the expression data was determined using one-way ANOVA, Tukey’s HSD test and the fold change, for which the null hypothesis was that no difference existed among the 3 groups. The FDR was controlled by adjusting the *P* value using the Benjamini-Hochberg algorithm. NimbleScan v2.5 software was used for quantification and image analysis of mRNA data. R script was used for all other analytical processes.

**Identification of DNA binding proteins.** The biotin-labeled DNA fragment of the *cydAB* promoter region was affixed to streptavidin-conjugated Dynabeads (Invitrogen) and then incubated with *V. vulnificus* cytoplasmic extract. Non-adhering and low-specificity DNA-binding proteins were removed by repeated washing with buffers that had non-specific DNA and a low salt concentration (22 mM pH 7.5 Tris-HCl, 4.4 mM EDTA, 8.9% sucrose, 62 mM NaCl, 0.3% protease inhibitor, 0.04% phosphatase inhibitor, 10 mM HEPES, 5 mM CaCl_2_, 50 mM KCl, 12% glycerol). Specific DNA-binding proteins were eluted by a buffer with a high salt concentration (0.5M NaCl). Single protein bands were cut from the SDS-PAGE gel for MALDI-TOF mass spectrometry analysis.

**LacZ reporter construction and β-Galactosidase assay.** The promoter-lacZ fusions were constructed by cloning the PCR Fragments containing the promoter sequence of target genes into pTL61T plasmid. The resulting recombinant plasmids were transformed into V. vulnificus by conjugation as described above. The screened reporter strains were grown in 2.5% NaCl HI broth under aerobic, microaerobic or anaerobic conditions. Cells were collected at appropriate time intervals and β-Galactosidase activity of the reporter strain was assayed. All the assays were performed in triplicates.

**Intracellular ATP level determination.** Intracellular ATP level of bacteria was measured by using the ATP Determination kit (Invitrogen, Eugene, OR) for quantitative determination of ATP with recombinant firefly luciferase and its substrate D-luciferin in accordance with the manufacturer’s protocol. Mid-logarithmic growth phase cells from each culture condition were used for the assay. Briefly, collected bacterial cultures (less than 10% of the total assay volume) were mixed with freshly made standard reaction solution containing firefly luciferase and its substrate D-luciferin, luminescence was detected by luminometer (Micro Lumat Plus LB 962, Berthold, Germany). ATP concentration was calculated from the standard curve generated with control ATP sample after subtracting the culture supernatant luminescence and was normalized to bacterial cell density OD600.

**Supplementary Discussion**

**Characterization of FexA-regulon**

In addition to *cydAB*, we also found that multiple genes that are potentially associated with redox regulation were under FexA regulation (Table 1). Mutation of VV1_1600-VV1_1601 encoding oxaloacetate decarboxylase, and VV1_3117 encoding the Na^+^/H^+^ antiporter NhaC, had no effects on *V. vulnificus* growth and motility (data not shown and Table 1), although oxaloacetate decarboxylase and the sodium motive force were reported to be associated with bacterial motility [3, 4]. Mutation of two gene clusters, VV2_0293-VV2_0295 and VV2_0297-VV2_0298 also had no effects on *V. vulnificus* growth and virulence (data not shown and Table 1). Moreover, mutation of VV1_0555-VV1_0556, which encodes glutamate synthase that catalyzes the conversion between glutamine and glutamate only slightly retarded growth in minimal medium (data not shown and Table 1). Interestingly, we found that the addition of glutamate to minimal medium profoundly improved the aerobic growth of the Δ*fexA* mutant (data not shown and Table 1). In *E. coli*, glutamate was reported to contribute to the proton motive force via consumption of intracellular protons and glutamate/GABA antiport when *cydB* was mutated and to stimulate the growth of the *cydB* mutant when supplemented in medium [5]. Given the downregulated *cydAB* and glutamate synthase, we speculate that the potential mechanism of glutamate in stimulating Δ*fexA* mutant growth might also contribute to the proton motive force. The genes involved in the glutamate/GABA shunt were not affected by the FexA mutation. Arginine and proline, which can be reversibly converted to glutamate, could also stimulate the growth of the Δ*fexA* mutant (data not shown and Table 1). The gene cluster VV2_0739-V2_0742, which encodes enzymes for polyhydroxyalkanoic acids (PHAs) biosynthesis, is reported for the first time to be regulated by an ArcA ortholog. PHAs are synthesized from acetyl-CoA and significantly accumulate under oxygen limitation. Generally, PHAs are regarded to be carbon and energy reserve materials. The fate of acetyl-CoA in bacteria depends on the environmental conditions; acetyle-CoA may be oxidized via the TCA cycle or can serve as a substrate for PHA synthesis. Under oxygen limitation, the TCA cycle enzymes citrate synthase and isocitrate dehydrogenase are inhibited by NADH accumulation, and acetyl-CoA no longer enters the TCA cycle but is instead converted to acetoacetyl-CoA by 3-ketothiolase, the first enzyme of the PHA biosynthetic pathway [6]. Thus, regulation of PHA biosynthesis by the oxygen-responsive regulator FexA appears to be quite probable. Mutation of the PHA gene cluster had no effects on *V. vulnificus* growth in nutrient HI broth and cytotoxicity but slightly attenuated mouse lethality by a 5-fold increase in LD_50_ and retarded growth in a minimal medium (data not shown and Table 1). PHAs seem to have some impact on *V. vulnificus* multiplication under nutrient-limited conditions and in vivo virulence expression.

**Putative regulation of FexA/CydAB axis**

CydAB plays an essential role in *V. vulnificus* survival, and the *cydAB* expression was profoundly affected by the FexA deletion regardless of oxygen availability. By contrast, in *E. coli*, maximal *cydAB* expression was observed under microaerobic conditions due to the activating function of ArcA; however, histone-like protein H-NS repressed *cydAB* expression under aerobic conditions, and FNR counteracted ArcA activation by repressing *cydAB* transcription under anaerobic conditions [7, 8, 9]. We speculate that the two putative FexA binding sites might have differential roles in activating *cydAB* transcription since compensating point mutations were clustered in only one FexA binding site. The *cydAB* promoter region has multiple poly AT stretches, which contribute to DNA bending and H-NS binding [10]. As shown in Fig. 6, P_cyd_SM1 bound H-NS, while P_cyd_WT did not. The SeqA binding GATC methylation site was discovered in only one site. The palindromic putative HexR binding sequence (ATGTTG-N_8_-CAACAT) appeared to overlap the second putative LeuO binding site. In an *E. coli* SELEX study, it was shown that each promoter was regulated by more transcription factors than experimentally recognized [11]. In the P_cyd_ promoter region, at least five proteins appeared to be directly related to transcription regulation: FexA, LeuO, HexR, SeqA and H-NS. LeuO is well documented to be an important anti-silencing factor overcoming H-NS mediated transcription repression in Gram-negative bacteria [12]. The reason why H-NS binds to P_cyd_SM1 may be related to the decreased binding of LeuO. If this happened when P_cyd_SM1 was expressed *in vivo*, the H-NS-bound promoter should have positively contributed to transcription. H-NS was reported to interact with mRNA in the AT-rich -40 to -25 region to enhance translation of genes with suboptimal ribosome binding sites [13]. In the P_cyd_ promoter, the archetypical Shine-Dalgarno sequence was not observed and the -40 to -35 region was AT-rich, suggesting a probable positive contribution of H-NS to the expression of β-galactosidase reporters. These results suggest that the FexA protein directly binds to the P_cyd_ promoter and counteracts probable transcriptional repressors to promote expression. In the absence of FexA, compensatory single nucleotide mutations, such as P_cyd_SM1, which confers a lowered binding affinity to transcription repressors, lead to the expression of crucial survival genes.

**Supplementary Tables**

**Supplementary Table 1. Bacterial strains and plasmids used in this study**

| **Strains and plasmids** | **Description** | **Source or reference** |
| --- | --- | --- |
| ***Vibrio vulnificus*** | |  |
| CMCP6 | Wild-type strain, clinical isolate |  |
| ∆*fexA* | CMCP6 with *fexA* deletion mutation | This study |
| ∆*lacZ* | CMCP6 with *lacZ* deletion mutation | This study |
| ∆*fexA*∆*lacZ* | CMCP6 with *fexA* and *lacZ* double deletion mutation | This study |
| ∆*fnr* | CMCP6 with *fnr* deletion mutation | This study |
| ∆*fexA(*c*fexA)* | *fexA* deletion mutation complemented with *fexA* gene | This study |
| ∆*fnr(*c*fnr)* | *fnr* deletion mutation complemented with *fnr* gene | This study |
| CydB R100H | CMCP6 with with a point mutation at the *cydB* gene(100AA, R to H) | This study |
| CydB G144A | CMCP6 with with a point mutation at the *cydB* gene(144AA, G to A) | This study |
| ∆2_0739-2_0742 | CMCP6 with gene cluster VV2_0739- 2_0742 deletion mutation | This study |
| ∆1_0449 | CMCP6 with gene VV1_0449 deletion mutation | This study |
| ∆1_0450 | CMCP6 with gene VV1_0450 deletion mutation | This study |
| ∆1_2767 | CMCP6 with gene VV1_2767 deletion mutation | This study |
| ∆1_2768 | CMCP6 with gene VV1_2768 deletion mutation | This study |
| ∆*fexA/*P_cyd_SM1 | *fexA* deletion mutation with a point mutation at the *cydAB* operon regulatory region (-117bp, A to C) | This study |
| ∆*fexA/*P_cyd_SM2 | *fexA* deletion mutation with a point mutation at the *cydAB* operon regulatory region (-109bp, C to A) | This study |
| ∆*fexA/*P_cyd_SM3 | *fexA* deletion mutation with a point mutation at the *cydAB* operon regulatory region (-109bp, C to T) | This study |
| ∆*fexA/*P_cyd_SM4 | *fexA* deletion mutation with a point mutation at the *cydAB* operon regulatory region (-105bp, A to G) | This study |
| ∆*fexA*∆*lacZ/*pP_cyd_SM1 | *fexA* and *lacZ* double deletion mutant harboring pTL61T::P_cyd_SM1 | This study |
| ∆*fexA*∆*lacZ/*pP_cyd_SM2 | *fexA* and *lacZ* double deletion mutant harboring pTL61T::P_cyd_SM2 | This study |
| ∆*fexA*∆*lacZ/*pP_cyd_SM3 | *fexA* and *lacZ* double deletion mutant harboring pTL61T::P_cyd_SM3 | This study |
| ∆*fexA*∆*lacZ/*pP_cyd_SM4 | *fexA* and *lacZ* double deletion mutant harboring pTL61T::P_cyd_SM4 | This study |
| ∆*fexA*∆*lacZ/*pP_cyd_WT | *fexA* and *lacZ* double deletion mutant harboring pTL61T::P_cyd_wild type | This study |
| ∆*lacZ/*pP_cyd_SM1 | *lacZ* deletion mutant harboring pTL61T::P_cyd_SM1 | This study |
| ∆*lacZ/*pP_cyd_SM2 | *lacZ* deletion mutant harboring pTL61T::P_cyd_SM2 | This study |
| ∆*lacZ/*pP_cyd_SM3 | *lacZ* deletion mutant harboring pTL61T::P_cyd_SM3 | This study |
| ∆*lacZ/*pP_cyd_SM4 | *lacZ* deletion mutant harboring pTL61T::P_cyd_SM4 | This study |
| ∆*lacZ/*pP_cyd_WT | *lacZ* deletion mutant harboring pTL61T::P_cyd_wild type | This study |
| P_cyd_SM1 | CMCP6 with a point mutation at the *cydAB* operon regulatory region (-117bp, A to C) | This study |
| P_cyd_SM2 | CMCP6 with a point mutation at the *cydAB* operon regulatory region (-109bp, C to A) | This study |
| P_cyd_SM3 | CMCP6 with a point mutation at the *cydAB* operon regulatory region (-109bp, C to T) | This study |
| P_cyd_SM4 | CMCP6 with a point mutation at the *cydAB* operon regulatory region (-105bp, A to G) | This study |
| ***Escherichia coli*** | | |
| DH5α | F^-^ *recA1*; restriction negative | ATCC |
| SY327 λ pir | Δ(*lac pro*) *argE* (Am) *rif nalA* *recA56* λ pir lysogen; host for π-requiring plasmids | [14] |
| SM10 λpir | *thi thr leu tonA lacY supE recA*::RP4-2-TcR::Mu | [14] |
| Plasmids | | |
| pCR2.1 | PCR TOPO TA cloning vector | Invitrogen |
| pDM4 | A suicide vector with ori R6K *sacB* and *Cm*^r^ | [14] |
| pLAFR3II | pLAFR3 with bla instead of cos site | [15] |
| pRK2013 | IncP, Km^r^, Tra Rk2^+^ repRK2 repE1 | [16] |
| pTL61T | Promoterless cloning vector; Ap^r^ | [17] |
| pTL61T::P_cyd_WT | DNA fragment containing *cydAB* operon regulatory region cloned into pTL61T | This study |
| pTL61T::P_cyd_SM1 | DNA fragment containing *cydAB* operon regulatory region with a point mutation at the  (-117bp, A to C) cloned into pTL61T | This study |
| pTL61T::P_cyd_SM2 | DNA fragment containing *cydAB* operon regulatory region with a point mutation at the  (-109bp, C to A) cloned into pTL61T | This study |
| pTL61T::P_cyd_SM3 | DNA fragment containing *cydAB* operon regulatory region with a point mutation at the  (-109bp, C to T) cloned into pTL61T | This study |
| pTL61T::P_cyd_SM4 | DNA fragment containing *cydAB* operon regulatory region with a point mutation at the  (-105bp, A to G) cloned into pTL61T | This study |

*Cm^r^*, Chloramphenicol resistance; *Km^r^*, Kanamycin resistance; Ap*^r^*, Ampicillin resistance

**Supplementary Table 2.** **Primers used in this study**

| **Primer** | **Sequence** |
| --- | --- |
| Deletion mutant construction for *fexA* | |
| *fexA*-1 | 5’-ACTAGTATCACCCCTTCCCAATCATT-3’ |
| *fexA*-2 | 5’-GTCGACTGGCGGTACCTAAATTTGTGA-3’ |
| *fexA*-3 | 5’-GTCGACGCGCACCGCATAAACAAAAA-3’ |
| *fexA*-4 | 5’-GGGCCCGTACTATCGCAAACACTATA-3’ |
| ∆*fexA* complementation | |
| *fexA*-5 | 5’-TTATAAGCTTCCACATCAAACATGCGGCT-3’ |
| *fexA*-6 | 5’-AGCTGGATTCTTACGCTTCTAAATCACCAC-3’ |
| Cloning of *cydAB* operon regulatory region | |
| Pcyd-F | 5’-GAATTCAGTTTGCAAGGCTAGGCATT-3’ |
| Pcyd-R | 5’-GGATCCGGTAACTCCTATGTGTCGGC-3’ |
| Deletion mutant construction for *fnr* | |
| *fnr*-1 | 5’-TGAACTAGTCAGAAAGGCCAGTACGCCCC -3’ |
| *fnr*-2 | 5’-AAGGTTGTTATGTAAATCTCTACTGCGACGTAGC-3’ |
| *fnr*-3 | 5’-AGTAGAGATTTACATAACAACCTTTCACTATTTG-3’ |
| *fnr*-4 | 5’-AGCGGGCCCGTGGTCTCTAACTCCTTAAG -3’ |
| Construction of point mutation in *cydAB* operon promoter region | |
| P_cyd_SM1-1 | 5’-GTTAAGCATCAGCGAATATAGCTGCGGC-3’ |
| P_cyd_SM1-2 | 5’-GCCGCAGCTATATTCGCTGATGCTTAAC-3’ |
| P_cyd_SM2-1 | 5’-GTTAATTGTTAATCATCAGCTAATATAG-3’ |
| P_cyd_SM2-2 | 5’-CTATATTAGCTGATGATTAACAATTAAC-3’ |
| P_cyd_SM3-1 | 5’-GTTAATTGTTAAACATCAGCTAATATAG-3’ |
| P_cyd_SM3-2 | 5’-CTATATTAGCTGATGTTTAACAATTAAC-3’ |
| P_cyd_SM4-1 | 5’-GGTTAGTTAATTGCTAAGCATCAGCTA -3’ |
| P_cyd_SM4-2 | 5’-TAGCTGATGCTTAGCAATTAACTAACC -3’ |
| P_cyd_SM5-1 | 5’-TAATATAGCTG GGATAATACTACTGCT-3’ |
| P_cyd_SM5-2 | 5’-AGCAGTAGTATTATCCGCAGCTATATTA-3’ |
| P_cyd_SM6-1 | 5’-GCATCAGCTAATATCGCTGCGGCTAATA-3’ |
| P_cyd_SM6-2 | 5’-TATTAGCCGCAGCGATATTAGCTGATGC-3’ |
| P_cyd_SM7-1 | 5’-TAAGCATCAGCTAACATAGCTGCGGCTA-3’ |
| P_cyd_SM7-2 | 5’-TAGCCGCAGCTATGTTAGCTGATGCTTA-3’ |
| P_cyd_SM8-1 | 5’-GTTAAGCATCAGCGAATATCGCTGCGGCTAATAC-3’ |
| P_cyd_SM8-2 | 5’-GTATTAGCCGCAGCGATATTCGCTGATGCTTAAC-3’ |

**Supplementary Table 3.** **Effect of mutations on the lethality of *V. vulnificus* in mice.**

| **Strain** | **LD_50_**  **(Fold increase in LD_50_: Mutant/Wild type)** | | |
| --- | --- | --- | --- |
|  | **Intraperitoneal LD_50_ Normal mice** | | **Intragastric LD_50_**  **Sucking mice** |
| Wild type | 4.0 X 10^5^ | | 4.0 X 10^6^ |
| ∆*fexA* | 1.0 X 10^7^  (25) | | 3.0 X 10^8^  (75) |
| ∆*fnr* | 2.1 X 10^6^  (5) | | 9.3 X 10^6^  (2.3) |
|  |  |  |  |

**Supplementary References**

1. Duong-Nu TM, Jeong K, Hong SH, et al. All Three TonB Systems Are Required for Vibrio vulnificus CMCP6 Tissue Invasiveness by Controlling Flagellum Expression. Infection and immunity. 2016 Jan;84(1):254-65. doi: 10.1128/IAI.00821-15. PubMed PMID: 26527216; PubMed Central PMCID: PMC4693995.

2. Kim SY, Thanh XT, Jeong K, et al. Contribution of six flagellin genes to the flagellum biogenesis of Vibrio vulnificus and in vivo invasion. Infection and immunity. 2014 Jan;82(1):29-42. doi: 10.1128/IAI.00654-13. PubMed PMID: 24101693; PubMed Central PMCID: PMC3911872.

3. Granjon T, Maniti O, Auchli Y, et al. Structure-function relations in oxaloacetate decarboxylase complex. Fluorescence and infrared approaches to monitor oxomalonate and Na(+) binding effect. PLoS One. 2010 Jun 3;5(6):e10935. doi: 10.1371/journal.pone.0010935. PubMed PMID: 20543879; PubMed Central PMCID: PMC2881705.

4. Kojima S, Yamamoto K, Kawagishi I, et al. The polar flagellar motor of Vibrio cholerae is driven by an Na+ motive force. J Bacteriol. 1999 Mar;181(6):1927-30. PubMed PMID: 10074090; PubMed Central PMCID: PMC93596.

5. Shepherd M, Sanguinetti G, Cook GM, et al. Compensations for diminished terminal oxidase activity in Escherichia coli: cytochrome bd-II-mediated respiration and glutamate metabolism. J Biol Chem. 2010 Jun 11;285(24):18464-72. doi: 10.1074/jbc.M110.118448. PubMed PMID: 20392690; PubMed Central PMCID: PMC2881772.

6. Anderson AJ, Dawes EA. Occurrence, metabolism, metabolic role, and industrial uses of bacterial polyhydroxyalkanoates. Microbiol Rev. 1990 Dec;54(4):450-72. PubMed PMID: 2087222; PubMed Central PMCID: PMC372789.

7. Cotter PA, Melville SB, Albrecht JA, et al. Aerobic regulation of cytochrome d oxidase (cydAB) operon expression in Escherichia coli: roles of Fnr and ArcA in repression and activation. Molecular microbiology. 1997 Aug;25(3):605-15. PubMed PMID: 9302022.

8. Govantes F, Albrecht JA, Gunsalus RP. Oxygen regulation of the Escherichia coli cytochrome d oxidase (cydAB) operon: roles of multiple promoters and the Fnr-1 and Fnr-2 binding sites. Molecular microbiology. 2000 Sep;37(6):1456-69. PubMed PMID: 10998176.

9. Tseng CP, Albrecht J, Gunsalus RP. Effect of microaerophilic cell growth conditions on expression of the aerobic (cyoABCDE and cydAB) and anaerobic (narGHJI, frdABCD, and dmsABC) respiratory pathway genes in Escherichia coli. Journal of bacteriology. 1996 Feb;178(4):1094-8. PubMed PMID: 8576043; PubMed Central PMCID: PMC177770.

10. Koo HS, Wu HM, Crothers DM. DNA bending at adenine . thymine tracts. Nature. 1986 Apr 10-16;320(6062):501-6. doi: 10.1038/320501a0. PubMed PMID: 3960133.

11. Ishihama A, Shimada T, Yamazaki Y. Transcription profile of Escherichia coli: genomic SELEX search for regulatory targets of transcription factors. Nucleic Acids Res. 2016 Mar 18;44(5):2058-74. doi: 10.1093/nar/gkw051. PubMed PMID: 26843427; PubMed Central PMCID: PMCPMC4797297.

12. Stoebel DM, Free A, Dorman CJ. Anti-silencing: overcoming H-NS-mediated repression of transcription in Gram-negative enteric bacteria. Microbiology. 2008 Sep;154(Pt 9):2533-45. doi: 10.1099/mic.0.2008/020693-0. PubMed PMID: 18757787.

13. Park HS, Ostberg Y, Johansson J, et al. Novel role for a bacterial nucleoid protein in translation of mRNAs with suboptimal ribosome-binding sites. Genes Dev. 2010 Jul 1;24(13):1345-50. doi: 10.1101/gad.576310. PubMed PMID: 20595230; PubMed Central PMCID: PMCPMC2895194.

14. Miller VL, Mekalanos JJ. A novel suicide vector and its use in construction of insertion mutations: osmoregulation of outer membrane proteins and virulence determinants in Vibrio cholerae requires toxR. Journal of bacteriology. 1988 Jun;170(6):2575-83. PubMed PMID: 2836362; PubMed Central PMCID: PMC211174.

15. Kim SY, Lee SE, Kim YR, et al. Regulation of Vibrio vulnificus virulence by the LuxS quorum-sensing system. Molecular microbiology. 2003 Jun;48(6):1647-64. PubMed PMID: 12791145.

16. Ditta G, Stanfield S, Corbin D, et al. Broad host range DNA cloning system for gram-negative bacteria: construction of a gene bank of Rhizobium meliloti. Proceedings of the National Academy of Sciences of the United States of America. 1980 Dec;77(12):7347-51. PubMed PMID: 7012838; PubMed Central PMCID: PMC350500.

17. Linn T, St Pierre R. Improved vector system for constructing transcriptional fusions that ensures independent translation of lacZ. Journal of bacteriology. 1990 Feb;172(2):1077-84. PubMed PMID: 2137119; PubMed Central PMCID: PMC208539.
